# Underground Water Extraction Using Synthetic Trees

**DOI:** 10.1021/acs.langmuir.6c01939

**Published:** 2026-08-12

**Authors:** Danielle N. Miller, Ndidi L. Eyegheleme, Cassie L. Freedlander, Jonathan B. Boreyko

**Affiliations:** Department of Mechanical Engineering, 1757 Virginia Tech, Blacksburg, Virginia 24061, United States

## Abstract

Unlike traditional well pumping from bulk reservoirs, trees can extract and elevate water spontaneously from moist soil. Here, we demonstrate transpiration-driven underground water harvesting by burying the bottom of a synthetic tree in water-saturated soil while encasing the nanoporous leaf within a condensing still. Evaporation from the leaf facilitates the continual transport of water up the tree’s tubes, with a cold plate on the condensing still converting the vapor back to liquid. A hydraulic model balances the negative Laplace pressure generated within the leaf against the hydrostatic pressure and viscous losses in the leaf and soil. We show that the evaporation-limited transpiration rate, and by extension the water harvesting rate, can be directly tuned through controlled heat inputs.

## Graphical abstract

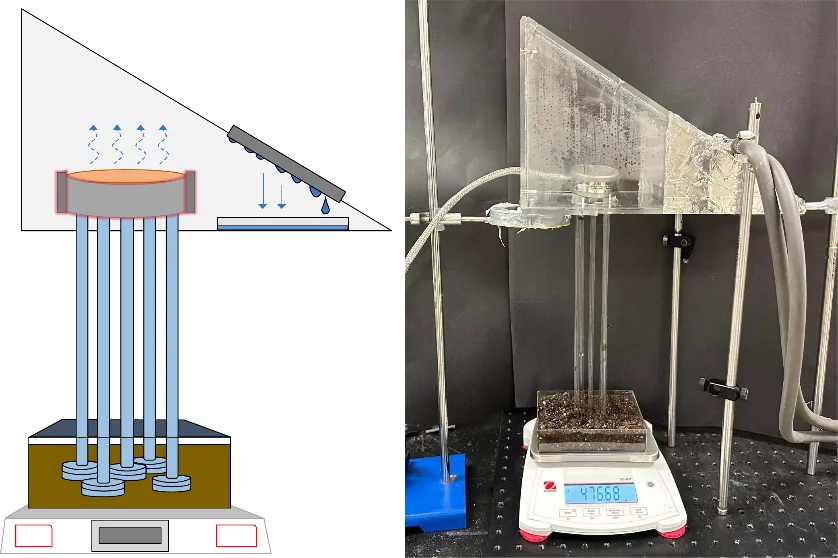

## Introduction

Clean water scarcity affects around 55% of the global population at least one month every year, and this number is expected to continue to rise.^1^ Potable water is often obtained by extracting groundwater from aquifers. The most traditional method of groundwater extraction is the dug well, in which a wide, shallow shaft is excavated until it intersects the water table; water is then raised manually with a bucket or by using a shallow pump.^2–4^ Driven wells consist of a narrow pipe driven directly into soft soil, with a screened tip to limit sediment intake.^3,4^ Drilled wells, constructed with percussion or rotary drills, are typically used to access deeper aquifers and are more costly to install and operate.^2–4^ Infiltration galleries employ perforated pipes buried beneath or alongside surface water bodies to collect naturally filtered seepage through permeable ground before pumping.^2^ Artesian wells and spring developments also provide access to groundwater, occurring when subsurface pressure within a confined aquifer drives water upward naturally or when the water table is exposed to the land surface, respectively.^2,5^

Each groundwater extraction method presents inherent constraints. Dug wells are highly susceptible to surface runoff contamination.^3,6^ Driven wells frequently experience clogging and contamination due to sediment intrusion.^6,7^ Drilled wells require a durable casing to maintain structural integrity and prevent collapse.^2,3^ Infiltration galleries are highly dependent on local groundwater and surface water conditions, resulting in variable yield.^2^ More generally, these engineered systems require external energy to lift water to the surface. Dug wells, driven wells, and infiltration galleries commonly rely on jet or centrifugal pumps to draw water upward, while drilled wells typically use submersible pumps to push water to the surface from greater depths.^3,4^ Although artesian wells and springs generally do not require external energy input, their availability and flow rates depend entirely on geological and seasonal factors that can vary over time.^8,9^

Plants use transpiration to transport water over great distances without external energy input.^10–16^ Synthetic trees replicate this process, passively pumping fluids by virtue of the negative Laplace pressure generated by nanoporous evaporators.^17–23^ For a vertically oriented synthetic tree, one or more conduits (i.e., “xylem”) are submerged in a liquid reservoir on their bottom ends, with their top ends embedded within nanoporous media serving as leaves. After prefilling the synthetic tree with degassed water, concave menisci form within the nanopores of the leaves during evaporation. This generates the negative Laplace pressure that establishes a hydraulic gradient from the evaporative interface, down the xylem-like conduits, to the roots or bulk reservoir.^11,17,24–31^ To date, synthetic trees have been shown to extract water or oil from bulk liquid reservoirs.^20,21,32,33^ Here, we demonstrate that synthetic trees can draw water from the interstitial spaces between saturated soil particles. Additionally, we show that evaporation-limited transpiration rates can be modulated through controlled heat input and that the vapor can be harvested using an above-ground condensing still.

## Results and Discussion

Figure 1 provides a schematic representation of the core components of the synthetic tree system. The structure consists of an array of five vertical tubes embedded halfway into a nanoporous artificial leaf. Upon saturation, liquid water fills the nanopores, forming concave menisci at the evaporative interface. As evaporation occurs at the leaf surface, the resulting capillary tension continuously pulls water upward in a hydraulically connected column. To exclude soil particles from the upward flow, filter caps were added to the base of each tube. Each cap consists of a 1 μm stainless steel mesh sandwiched between two rigid flanges. In natural soils, the smallest particles, clay, can be as small as 2 μm.^34^ Although loamy soil contains a fraction of clay particles, it is predominantly composed of larger silt and sand grains as well as some organic matter.^35^ The 1 μm mesh therefore prevents even the smallest soil particles from passing into the system. The filter caps are buried in soil whose interstitial spaces were fully saturated with water. This ensures a continuous liquid water phase between the moist soil and the synthetic tree. Though the matric potential of both a pure liquid reservoir and fully saturated soil are both approximately zero, meaning there is negligible capillary suction, there is nonetheless a hydraulic resistance caused by viscous Darcy flow across the soil’s porous media that does not exist in pure liquid reservoirs.

**Figure 1:**
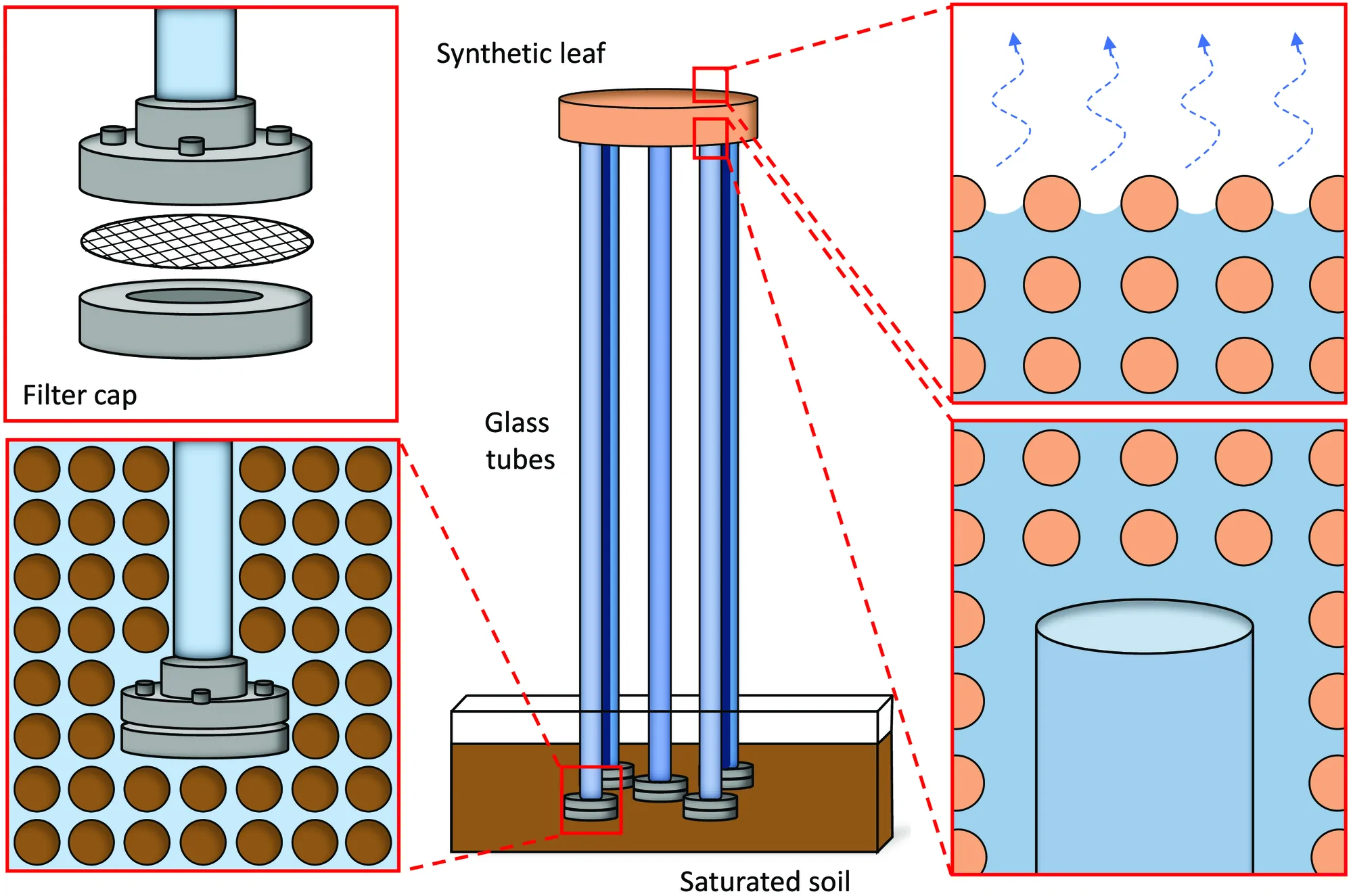
Schematic of a partially buried synthetic tree system. The ends of glass conduits are fitted with filter caps that screen particulate matter from the incoming water. The top left inset depicts the 1 μm pore-size mesh sandwiched between two flanges. The bottom left inset shows the partially buried tube enclosed in fully saturated soil; water fills all interstitial spaces between soil particles. The top right inset shows the concave nanomenisci within the ceramic disk that generate the Laplace pressure via evaporation. The bottom right inset shows the top of the tube embedded into the ceramic disk and the resulting continuity of water.

Figure 2 illustrates the full experimental setup for the partially buried synthetic tree for underground water extraction. This setup includes three additional components not depicted in Figure 1: a mass balance to quantify transpiration over time, a band heater wrapped around the leaf to control the evaporation rate, and a condensing still to collect the evaporated water. A container was prefilled with wetted soil, with the water level maintained flush with the soil’s top to ensure that all interstitial pore spaces were saturated. The soil reservoir was placed on a mass balance with a readability of 0.01 g and a linearity of 0.03 g. Measurements were recorded at 1 min intervals for the duration of each trial (about 150 min). Each minute increment resulted in a mass change large enough to overcome the linearity variation, making each recorded value significant. For unheated cases where evaporation from the water in the soil may be comparable in magnitude to that from the nanoporous leaf, a Parafilm cover was placed over the soil to prevent mass loss unrelated to transpiration across the tree itself. By conservation of mass, the resulting mass loss then corresponded directly to water transpired from the leaf. The leaf was inserted into the condensing still through a hinged door on the bottom face, which was secured beneath the leaf, suspending it slightly from the floor. A band heater entered through a small port in the vertical face of the still, where the circular heating element surrounded and made light contact with the perimeter of the ceramic leaf. Power levels of 0 (i.e., off), 10, and 20 W were applied to systematically vary the transpiration rate. The water vapor within the condensing still was drawn to a cold plate maintained at 10 °C, where it recondensed and dripped into a collection plate located below that was only measured after the full extent of a trial. Although a substantial amount of vapor was condensed on the cold plate, condensation also frequently occurred on the interior walls of the still. In such cases, the residual liquid was carefully scraped from the walls into the collection tray prior to final mass measurements to achieve more accurate collection percentages.

**Figure 2:**
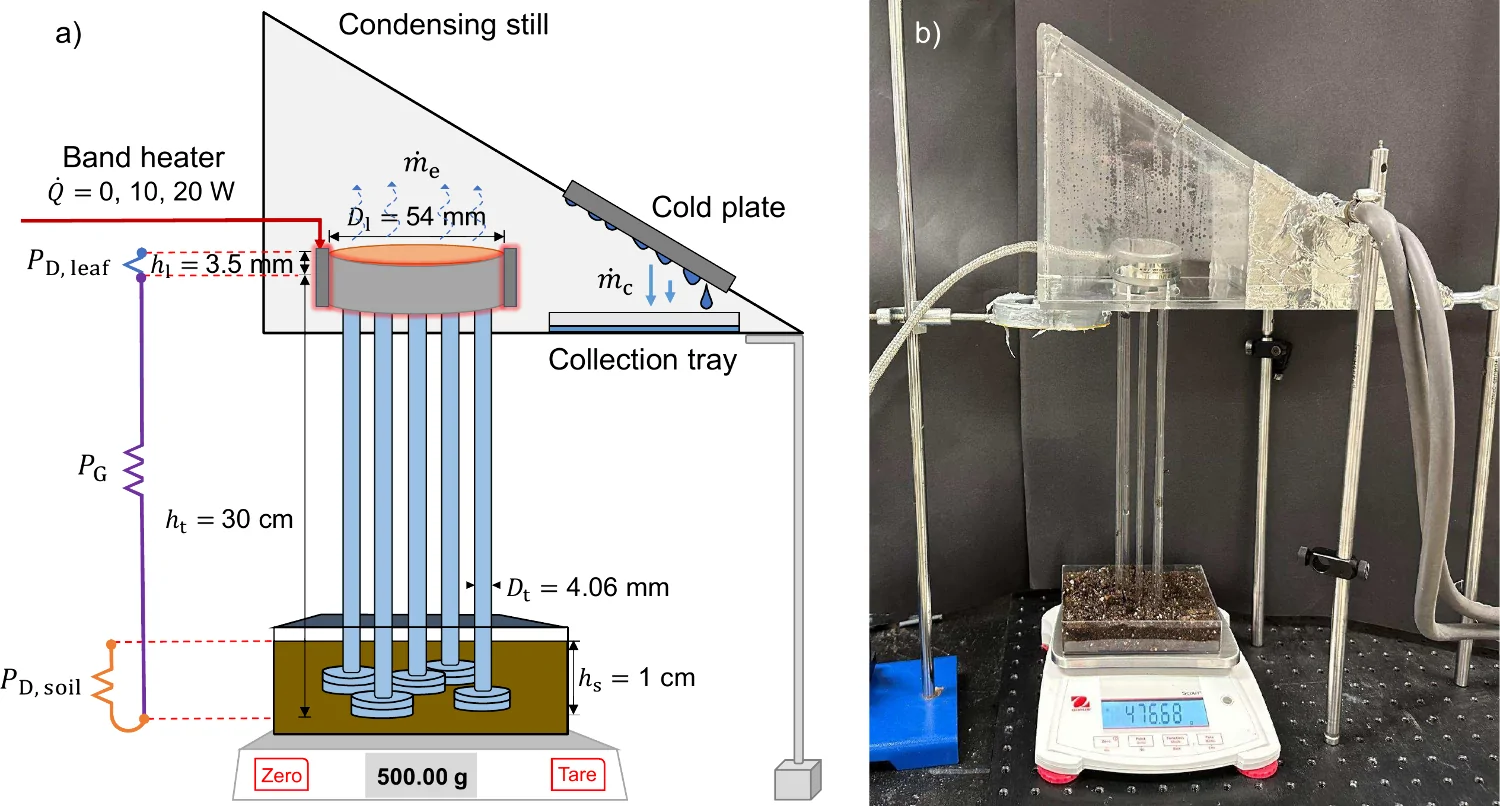
Underground water extraction and harvesting using a synthetic tree. (a) Schematic and (b) photograph of the experimental setup. The leaf is enclosed with a condensing still that uses a cold plate to harvest the water. The circuit to the left of the schematic illustrates the positive pressure drops across the soil and tree that collectively balance the negative Laplace pressure generated within the leaf. A resistive band heater was clamped around the perimeter of the leaf to vary the transpiration rate.

Figure 3a presents the time-normalized evaporation and water collection rates for each applied power input to the leaf. A higher applied heater power results in a greater evaporative flux, which in turn enhances the liquid flow rate up the tree. Specifically, the transpiration mass flow rates were $\overset{˙}{m_{e}}\approx 0.042\pm 0.011$ g/min without applied heating (i.e., pure diffusion), $\overset{˙}{m_{e}}\approx 0.25\pm 0.09$ g/min for Q̇= 10 W, and $\overset{˙}{m_{e}}\approx 0.30\pm 0.03$ g/min for Q̇= 20 W, where the uncertainty corresponds to one standard deviation. The water collection rates were $\overset{˙}{m_{c}}\approx 0.0024\pm 0.0002$ g/min for the unheated leaf, $\overset{˙}{m_{c}}\approx 0.12\pm 0.05$ g/min for Q̇= 10 W, and $\overset{˙}{m_{c}}\approx 0.113\pm 0.008$ g/min for Q̇= 20 W.

**Figure 3:**
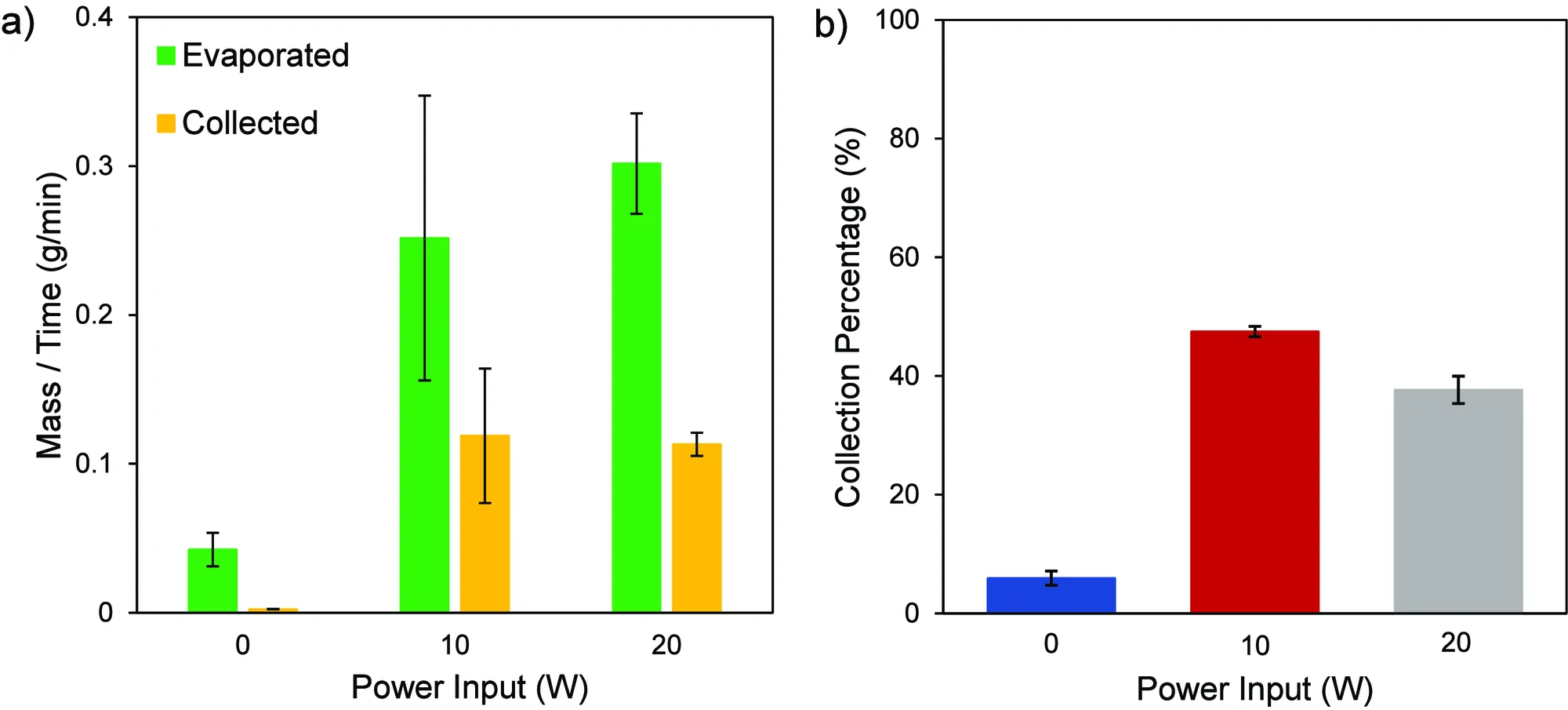
Plots showing the (a) evaporation and collection mass flow rates and (b) collection efficiencies of the synthetic tree-condensing still system with controlled power inputs of 0, 10, or 20 W to the band heater wrapped around the synthetic leaf. The error bars correspond to one standard deviation across three separate trials.

However, Figure 3b reveals that the collection efficiency, defined as the percentage of transpired water vapor that is recondensed and collected by the still, does not increase monotonically with power. Instead, the Q̇= 10 W condition exhibits the highest collection efficiency of η ≈ 47.5 ± 0.9%, followed by η ≈ 38 ± 2% at Q̇= 20 W and only η ≈ 5.9 ± 1.2% for Q̇= 0 W. The 10 W case has the highest trial-to-trial variability for the evaporation and collection mass flow rates; however, their ratio (i.e., collection percentage) was remarkably self-consistent. For the unheated case, if the still was perfectly sealed and all the vapor that was evaporated but not recondensed existed in the air, the supersaturation over the cold plate would be *S* = 171. The supersaturation is defined as the ratio of water vapor concentration within the condensing still to the saturation concentration, *S* = *C*/*C*_*sat*_. Here, the total vapor mass transpired into the still assuming perfect sealing (*m* = 6.83 g for the unheated case) was normalized by the volume of the still (*V* = 4252.5 cm^3^) to obtain the maximum possible vapor concentration, *C* = 1.606 kg/m^3^. This value is unrealistically large, indicating that there is enough leakage from the still to cause much of the water vapor to remain thermodynamically stable in the gas phase within the condensing still. At the other extreme of the 20 W condition, the supersaturation of the walls at 21 °C would have a value of *S* = 344. This overwhelms the design of the still itself, such that condensation can occur on the uncooled walls before reaching the cold plate, even with leakage simultaneously occurring. The best balance is struck at 10 W, where the vapor is sufficiently supersaturated to drive condensation on the cold plate even in the midst of leakage, but with less condensation occurring on the uncooled walls compared to the 20 W case.

Figure 4 details the resistive pressures that characterize flow through the synthetic tree system. The hydraulic load created by the negative Laplace pressure from the nanomenisci extends the full length of the tree and into the soil below, as noted in Figure 2. The Laplace pressure is therefore equal to the additive pressure drops across all individual components of the tree: viscous pressure drops through the wetted leaf, tubes, and soil, as well as a hydrostatic pressure drop in the tubes.

1 $$\left|P_{L}\right|=\Delta P_{\mathrm{D,}\mathrm{leaf}}+\Delta P_{P}+\Delta P_{G}+\Delta P_{\mathrm{D,}\mathrm{soil}}$$

where *P*_D, leaf_ is the Darcy pressure drop through the leaf, *P*_P_ and *P*_G_ are the Poiseuille and hydrostatic pressures through the tubes, and *P*_D, soil_ is the Darcy pressure drop through the soil. The viscous pressure drop through the synthetic leaf and soil can be calculated using Darcy’s Law,

2 $$\Delta P_{D}=\frac{Qh\mu }{\kappa A}$$

where Q is the volumetric flow rate, *h* is the height of the leaf or soil (*h*_l_ = 3.5 mm or *h*_s_ = 1 cm), μ is the viscosity of water, *A*_l_ = 22.9 cm^2^ and *A*_s_ = 64.7 mm^2^ are the cross-sectional areas of the leaf and soil, respectively, and κ is the intrinsic permeability of the porous media. In turn, the intrinsic permeability is given by $\kappa =\left(\varphi r_{pore}^{2}\right)/\left(8\tau \right)$ , where φ ≈ 0.32 is the manufacturer-estimated porosity of the nanoporous disk, *r*_pore_ ≈ 80 nm is the manufacturer-provided effective pore radius, and τ ≈ 3.5 is the estimated tortuosity typical of a three-dimensional porous network.^36,37^ This results in an intrinsic permeability of κ ≈ 7.3 × 10^–17^ m^2^ for the nanoporous synthetic leaf. A typical intrinsic permeability for the loamy soil used here is κ = 3.78 × 10^–13^ m^2^.^38^ The length scale from the surface of the soil to the inlet of the buried tubes is assumed to be approximately equivalent to the extent to which the pressure gradient extends across the soil, as water that is in close vicinity to the tree is pulled first and replenished by that above it. The effective flow area through the soil is equal to the combined area of all five tubes. The flow rates used in the calculations of Darcy pressure were the transpiration rates through the tree at each heat input, measured as *Q* = 7 ± 2 × 10^–10^ m^3^/s for the unheated leaf, *Q* = 4.2 ± 1.6 × 10^–9^ m^3^/s for the 10 W condition, and *Q* = 5.0 ± 0.6 × 10^–9^ m^3^/s for the 20 W condition.

**Figure 4:**
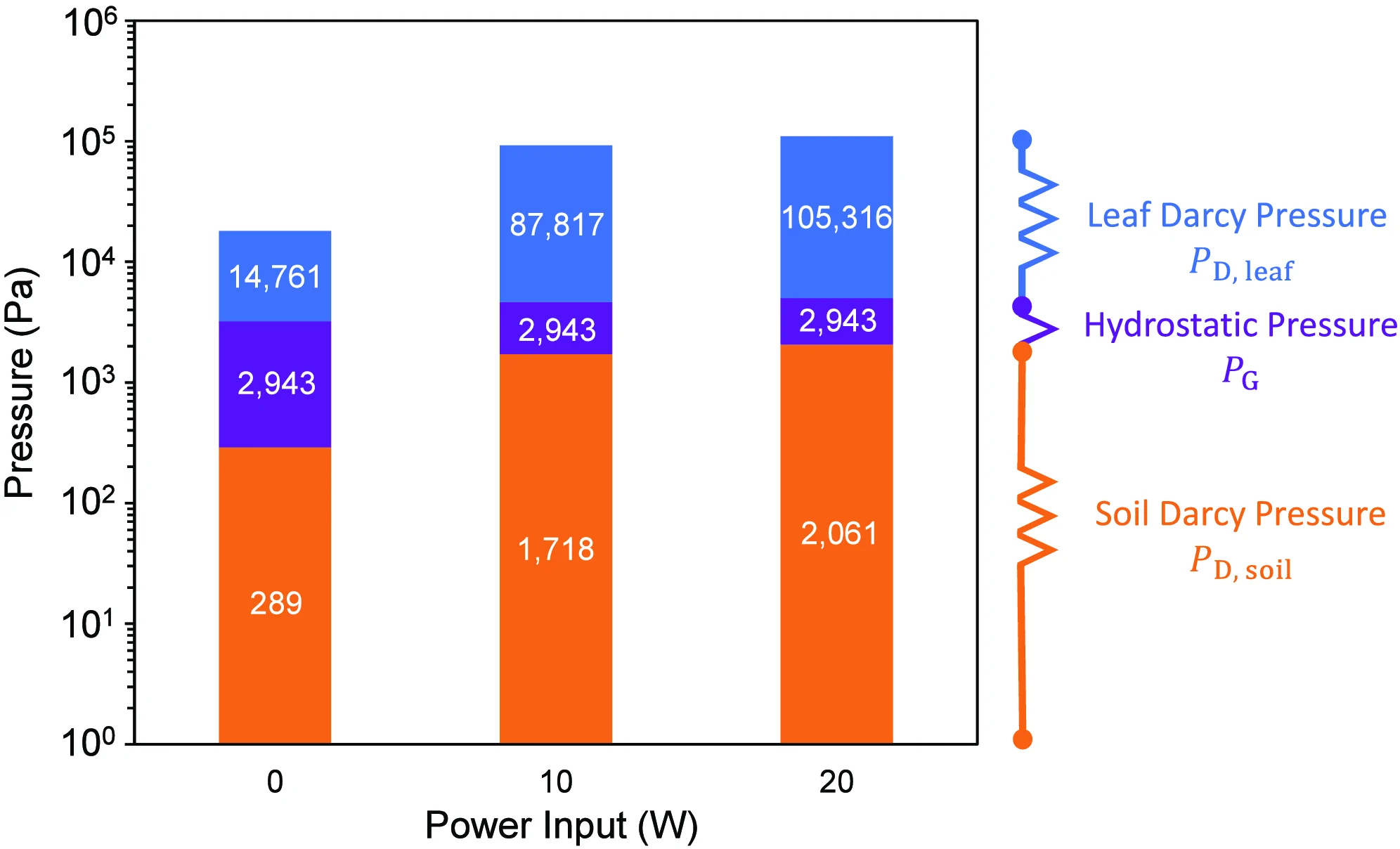
Constitutive positive pressures across the synthetic tree system for all power inputs. The three pressures in each stack combined are equivalent to the Laplace pressure generated at the evaporative interface driving the flow. A resistance circuit representative of the combined pressures can also be seen next to their respective tree components in Figure 2.

For any given transpiration rate *Q*, the viscous Poiseuille pressure drop along the tubes can be calculated as

3 $$\Delta P_{P}=\frac{8Qh_{t}\mu }{\pi Nr_{t}^{4}}$$

where *h*_t_ = 30 cm is the height of the tubes, *N* = 5 is the number of tubes, and *r*_t_ = 2.03 mm is the inner radius of the tubes. The height-to-diameter ratio of the tubes used in this work is much lower than that of natural trees since we attempt to maximize hydraulic conductance through the system for engineered applications. For the 20 W condition (i.e., highest flow rate), Δ*P*_P_ = 0.0453 Pa. This is at least 5 orders of magnitude smaller than the other competing pressures and is therefore considered negligible. The hydrostatic pressure, Δ*P*_G_ = *ρgh*_t_ = 2943 Pa, is constant for each trial, as it is based on the flow-independent tube height. The low permeability and appreciable thickness of the synthetic leaf result in large Darcy pressure drops of Δ*P*_D, leaf_ = 14,761 Pa, 87,817 Pa, and 105,316 Pa for the unheated leaf, 10 W condition, and 20 W condition, respectively. These Darcy pressures are even larger than the hydrostatic pressure inherent to holding the water against gravity, although this ratio can change dramatically based on the tree design and transpiration rate, as will be seen in the proceeding section. The soil Darcy pressures are Δ*P*_D, soil_ = 289 Pa, 1718 Pa, and 2061 Pa for the unheated leaf, 10 W condition, and 20 W condition, respectively. The Darcy pressure of the soil is on the same order as the hydrostatic pressure, due to a relatively larger permeability value compared to the nanoporous leaf.

Figure 5 is a collection of 3-dimensional plots that model the maximum depth that a synthetic tree can extend into the soil for a given leaf thickness, pore diameter, and transpiration flux. The Darcy pressure drop across the leaf is governed by the leaf thickness, pore size, and transpiration rate, while the tree height determines the hydrostatic pressure drop. The maximum allowable soil depth corresponds to the critical height where the Darcy pressure plus the hydrostatic pressure are equivalent to the maximum possible Laplace suction. Three leaf thicknesses are considered: 10 μm (Figure 5a), 100 μm (Figure 5b), and 3.5 mm (Figures 5c and d).

**Figure 5:**
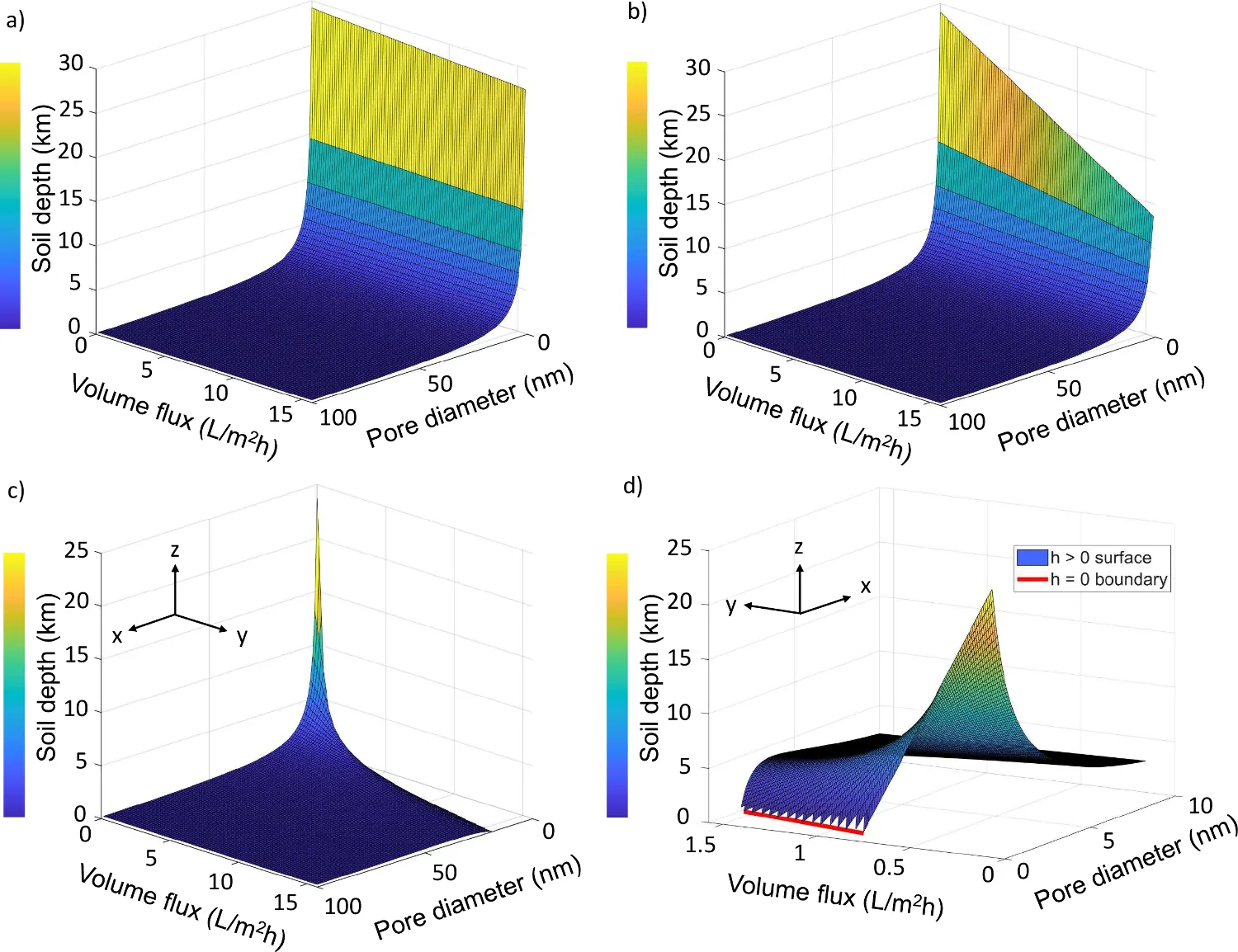
Three-dimensional plots of soil depth as a function of volume flux and pore diameter for leaf thicknesses of (a) 10 μm, (b) 100 μm, and (c) 3.5 mm. (d) Magnified portion of panel c, showing pore diameters from 0 to 10 nm and volume fluxes from 0 to 1.5 L/m^2^ h. Small leaf thicknesses minimize Darcy pressure and allow the hydrostatic pressure to dominate, facilitating taller trees. As leaf thickness increases, the Darcy pressure term becomes relevant until there is a direct competition between pressures; a crossover point is observed where Darcy pressure overwhelms the system, drastically decreasing the attainable soil depth. For practical applications, the maximum possible tree height should correspond to the minimum specified volume flux, rather than the upper limit of height, which corresponds to negligible volume flux.

Figures 5a and 5b are dominated by hydrostatic pressure within the tubes because the small leaf thicknesses minimize the Darcy pressure contribution. However, the effect of increasing leaf thickness becomes more apparent at high volume fluxes in Figure 5b, where the maximum soil depth begins to approach zero. In Figure 5c, the even larger leaf thickness produces a regime in which the Darcy pressure drop can dominate the hydrostatic over a wide parameter space. Indeed, facilitating an appreciable hydrostatic pressure column was only possible for the combined conditions of smaller pore diameters and lower fluxes. Increasing either the volume flux or pore diameter shifts the pressure balance toward Darcy pressure dominating. Figure 5d presents an inset of Figure 5c where the x and y axes are rotated and their limits confined to highlight the crossover between the two pressure regimes. The graph terminates at the red line corresponding to *h* = 0 km, showing only positive (physical) soil depths. A sharp decline in the curve occurs with increasing flow rate, marking the transition from a hydrostatic-dominated to a Darcy-dominated regime. In the Darcy-dominated regime, a decrease in pore diameter actually results in lower possible soil depths despite the stronger Laplace pressure, because the Darcy pressure increases nonlinearly. Realistic environmental soil can have a spatially varying permeability that would make the Darcy flow analysis more complex, requiring a numerical solution.

Across all graphs, the maximum soil depth is approximately 25 km or higher. By comparison, submersible pumps used to access deep aquifers typically operate at depths of around 45 m for residential wells and 240 m for industrial-grade applications, depending on pump power and well design.^2^ The maximum theoretical pumping depth of synthetic trees far surpasses that of existing energy intensive solutions, with the nuance that increasingly smaller volume fluxes are possible with increasing height.

For the commercially available nanoporous ceramic disks used in this study, the maximum disk diameter is *D* = 0.105 m. An underground water harvester with a total lateral extent of a square meter would therefore require about 10 trees, each embedded with approximately 10 tubes. This means a 50 m tall tree, comparable with the depth of residential wells, would require about 5,000 m of tubing, while a 250 m tall tree for industrial-grade applications would require about 25,000 m. Using commercially available ceramic leaves ($100/leaf), high-density polyethylene (HDPE) tubing ($3/m), standard tube fittings ($5/fitting), and stainless steel mesh for particulate filtration at the ends of the tubes ($10/m^2^), the estimated material cost is approximately $16,500 for a 50 m tree and $26,500 for a 250 m tree. However, we expect that next-generation synthetic trees will utilize custom-made leaves, rather than these small commercial ceramics. If 100 μm thick synthetic leaves were made via sintering industry-grade silica particles ($50/kg), the totals would be $15,500 for the 50 m tree and $25,500 for the 250 m tree. In both cases, the tubing itself accounts for over 95% of the total cost, but we expect the custom-made leaves will be more scalable for practical hydraulic systems. These estimates represent a broad, order-of-magnitude material cost analysis only and do not account for installation, maintenance, or replacement costs, which will ultimately influence the overall economic feasibility.

For practical applications of synthetic trees for groundwater extraction, several challenges remain. Over long-term operation, the soil filters could foul or clog and require replacement, or extra-small particulates could invade the tree itself and foul the leaves, reducing performance. In addition, the higher values of flow rates, and by extension, higher magnitudes of Laplace suction, would require the integration of waste heat or active heating sources, as purely diffusive evaporation into the air cannot achieve high evaporative fluxes. Future study could involve durability and scalability tests of the synthetic tree, as well as improvements to the condensing still design and construction to increase the overall efficacy and yield.

### Subsaturated Soil

In addition to fully saturated soil, trials were conducted using subsaturated soil partially wetted (50% of the void volume) to model more realistic soil conditions. These trials commenced similarly to the saturated condition; however, after a period of time, transpiration was inhibited by the incursion of air bubbles into the tubes. As shown in Figure 6a, these individual air bubbles eventually formed a plug that forced water in the tubes back into the reservoir beneath. This resulted in the “negative” evaporative mass flow shown in Figure 6b.

**Figure 6:**
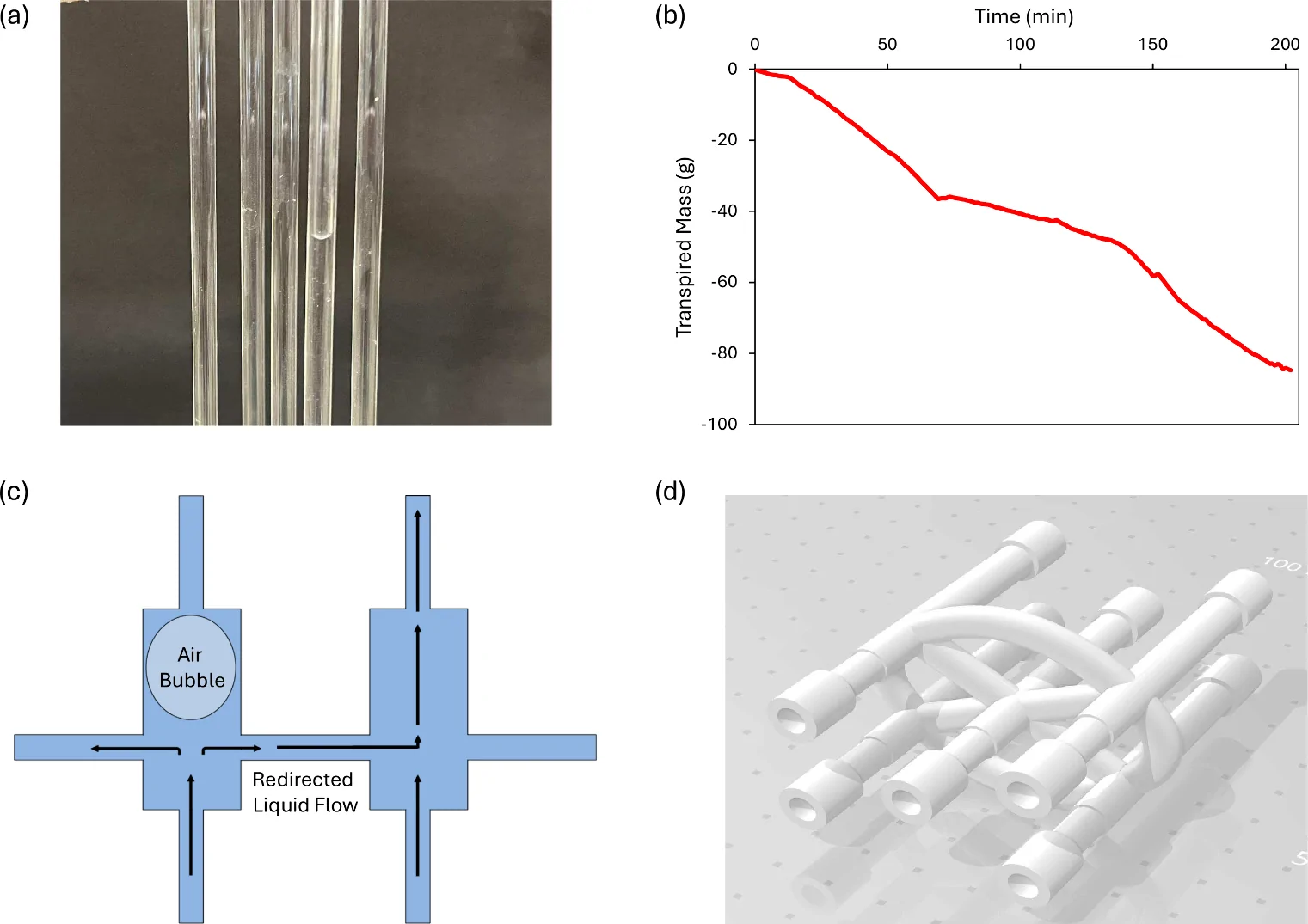
Summary of negative results with subsaturated soil. (a) Air plugs within tubes that forced water back into the reservoir. (b) Plot showing decreasing transpired mass over time, i.e., backwash of water from the tree into the underlying reservoir. (c) Schematic of the proposed solution with interconnections between all tubes, trapping air bubbles and redirecting fluid flow. (d) Model of a 3D-printed piece that was tested without success.

In order to mitigate this problem, a three-dimensional connective piece was designed to trap rising bubbles while providing alternative liquid pathways to prevent clogging (Figure 6c,d). The piece was 3D printed using a high-temperature resin to withstand the priming of the synthetic tree; it was placed between two 15 cm segments of glass tubes acting as xylem conduits and was sealed using a thermally resistant epoxy. Despite extensive iterative experiments and modifications of the design of the piece, the water within the connections and the underlying column was not able to be suspended successfully when connective tubes were present.

In addition to soil saturation, potential variance in soil temperature was unaccounted for in this work. For fully saturated conditions, the soil temperature would not primarily change the results, as the evaporation rate is at the leaf-level. However, it could secondarily change the water viscosity or the degree of soil saturation in subsaturated cases, which would affect the hydraulic resistance of the Darcy flow across the soil. Future research should determine approaches for synthetic transpiration to persist in realistic subsaturated soil conditions, as this is important for practical groundwater extraction applications.

## Conclusions

In summary, we have demonstrated that transpiration in a synthetic tree can be utilized for passive underground water harvesting. The pumpless underground ascent, evaporation, and collection of water using a synthetic tree and condensing still highlights the potential for nature-inspired water harvesting technologies that operate without external energy input. This work expands upon existing synthetic tree models by showing that the Laplace-pressure-generated hydraulic gradient can be sustained across the tree as well as through interstitial soil spaces, rather than requiring a bulk liquid reservoir. We distinguish between hydrostatic-dominated versus Darcy-pressure-dominated regimes, leading to the conclusion that the maximum pumping depth of a synthetic tree can significantly exceed that of conventional pumps, albeit at lower flow rates.

The addition of a controlled heat input to the synthetic leaf shows tunability of the transpiration rate, and consequently, the water collection rate. Previous works that tuned the hydraulic load and transpiration rate of synthetic trees only varied the overlying humidity or gas flow conditions.^17,32,33^ One work did utilize a solar lamp over the leaf,^21^ but only at a fixed intensity such that the transpiration rate was not tuned. While we demonstrate variable transpiration here through resistive heating, future implementations could be made renewable by utilizing waste heat or concentrated solar power. In the future, the condensing still design should be improved in order to boost the water collection rates, especially noting the need for near-perfect sealing to prevent leakage. The inadvertent crevices in the design caused leakage of water vapor, especially at the higher power inputs, that could have been recondensed and collected for use.

## Experimental Setup

### Synthetic Tree

The synthetic tree was fabricated by drilling five recesses of 7 mm diameter and 3.5 mm depth into a commercially available porous ceramic disk (Soilmoisture Equipment Corp, 0604D02-B15M1). This disk can be characterized by a porosity of ϕ ≈ 0.32, a pore radius of *r*_p_ ≈ 80 nm, a diameter of 54 mm, and a thickness of 7 mm. It is important to note that an effective thickness of 3.5 mm was used for Darcy pressure drop calculations (eq 2) to account for the height from the drilled recesses to the evaporative surface. Five borosillicate glass tubes with a height of 30 cm and an inner radius of 2.03 mm were then embedded into the drilled recesses and sealed using a heat and water resistant sealant. Filter caps, added to the bottom of each tube, were comprised of a 1 μm stainless steel mesh (TWP Inc., 400 × 2800TL0012) bolted between two stainless steel flanges.

### Condensing Still

A right triangular prism-shaped condensing still was constructed using acrylic sheets. The right-triangular cross section exhibited a base length of 31.5 cm and a height of 18 cm, with a width of 15 cm extending perpendicular to the cross section. A circular hole with a diameter of 5 cm, just under the diameter of the synthetic leaf, was cut into the bottom face of the still. This hole was bisected with a cut spanning the width of the still, and a hinge was added to create a floor-mounted door. A 1 cm diameter hole was added on the side face of the still to accommodate a band heater. A cold plate measuring 15 cm × 4 cm was added to the top face of the still and all pieces were bonded together using a heat and water resistant epoxy.

### Experimental Procedure

Prior to each experiment, the synthetic tree was primed by placing it in boiling water on a hot plate set at 260 °C for 4 h in order to remove all noncondensible gases from the system. This filled the tree completely with degassed water, preventing cavitation under negative pressure. After heating, the system was allowed to cool overnight while remaining within the water; a heavy lid remained on the pot for this period to minimize the diffusion of noncondensible gases back into the system. The next morning, filter caps were also dropped into the degassed water to eject air bubbles from the stainless steel meshing.

Three trials of each power input condition (0, 10, and 20 W) were conducted with the leaf encased in a condensing still and the lower ends of the tubes buried in fully saturated soil. In all cases, the primed synthetic tree was transferred from the pot to the experimental setup. While still submerged in the pot, a circular plug, 3D printed with high temperature resin and containing holes that matched the vertical tubes’ arrangement, was slipped over the ends of the tubes. Filter caps were then fitted onto the ends of the tubes. The tree was transferred from the pot to the test rig with the ends of the tubes remaining submerged in an intermediate container to limit the air exposure time. After placing the tree into the soil reservoir, the intermediate container was removed.

The synthetic leaf was inserted into the condensing still through an opening on the bottom face and was secured using a hinged door, while the resin plug rested against the bottom face to limit vapor loss. A circular band heater entered the still through a hole in the side face and was positioned such that its metal component made light contact with the perimeter of the ceramic disk. A cold plate was bonded to the top face of the still, and cold water maintained at 10 °C was circulated through the condenser to promote condensation within the chamber. A collection tray was located just below the cold plate within the still to capture the recondensed water.

Dry soil was mixed with water until saturated and then layered into the reservoir such that the ends of the tubes were buried halfway into the soil. Water was then added until level with the soil surface to ensure full saturation and hydraulic continuity. The reservoir was placed on a mass balance that recorded measurements at 1 min intervals for the duration of each trial.

During experimentation, a given power input influenced the transpiration rate through the synthetic tree. As flow occurred, the mass balance recorded the mass loss over time. Water vapor generated within the still frequently condensed on interior surfaces as well as the intended cold plate. At the conclusion of each trial, the collection tray was removed and weighed. Any additional condensation that accumulated on interior walls, particularly at higher power inputs, was carefully scraped into the collection tray before taking a final mass measurement to account for water collection.
